# Radiologists' perceptions and readiness for integrating artificial intelligence in diagnostic imaging: A survey-based study

**DOI:** 10.6026/9732063002001943

**Published:** 2024-12-31

**Authors:** Prasanna Sakthi Aravazhi, Kumaran Ottilingam Ravindran, Kanika Balasubramani, Mohammed Kamil, Kanishka Gouthaman, Lalit Karki, Sandhiya Thiyagarajan, Akshay Sureshkumar Nair

**Affiliations:** 1 Institute of Internal Medicine, Madras Medical College, Chennai, India; 2 Department of Radiology, Kathmandu Medical College, Kathmandu, Nepal; 3 Department of Internal Medicine, MMC, RGGGH, Chennai, India; 4 Department of General Medicine, Starcare Hospital, Calicut, India

**Keywords:** Artificial intelligence, diagnostic imaging, radiologists' perceptions, AI readiness, survey-based study, ethics in AI

## Abstract

Artificial intelligence (AI) is revolutionizing diagnostic imaging, enhancing precision, speed and efficiency. This study explored radiologists' perceptions of AI through a survey of 100 radiologists across various institutions, focusing on awareness, benefits, concerns and preparedness for AI adoption. Most radiologists recognized AI's potential to improve diagnostic accuracy and workflow efficiency but expressed concerns about its reliability, job displacement and ethical implications. Readiness to adopt AI varied significantly based on age, experience and familiarity with AI tools. These findings underscore the need for targeted education and training programs to address skepticism and support the effective integration of AI into diagnostic imaging practices.

## Background:

The innovation in healthcare has therefore led to AI becoming one of the most influential innovations made in healthcare, ranging from predictive analytics to personalized treatment planning and is now being used in diagnostic imaging for enhancing image analysis and optimizing workflow through better diagnostic accuracy. Through intricate algorithms and machine learning deep learning, AI can assist radiologists in quickening their analysis and possibly making more accurate diagnoses about the images through information that does not appear directly to the naked human eye [1, 2]. Diagnosis imaging with the integration of AI has promising beneficial impacts on the outcome of a patient by reducing errors from bad diagnoses, faster processing of images and giving radiologists more challenging challenges that need the expertise of the human eye [3]. However, integration of AI in diagnostic imaging has been adopted in radiology with varying levels of acceptance. Radiologists will be highly involved in image interpretation and the perception and acceptance of AI among them would be critical variables that would make or break the adoption and success of AI in clinical environments. However, AI as a "disruptive" technology can alter the current practice of diagnosis and such situations may raise questions about job displacement and ethical and technical issues of machine decision-making [4, 5]. The purpose of this study is to assess radiologists' preparedness for the adoption of AI-based technologies in the context of diagnostic imaging, including the perceived benefits and barriers. The researchers administered a survey to 100 radiologists from various institutions. These factors facilitating AI adoption among radiologists were examined based on age, years of experience and familiarity with AI technology. Therefore, it is of interest to inform the development of targeted educational initiatives and policies that will ease the transition from this traditional state to AI-enabled diagnostic imaging in radiology [6, 7].

## Methodology:

This study had a cross-sectional, survey-based design to assess the perceptions and preparedness of radiologists to accept AI in diagnostic imaging. A structured, self-administered questionnaire was specifically formulated based on an extensive review of the literature and consultation with experts to obtain perceptions, knowledge and perceived challenges in the context of AI in radiology.

## The questionnaire contained 25 items categorized into three sections:

Demographic and practice characteristics, perceptions of AI in diagnostic workflows and readiness for AI adoption. Questions regarding demographics included age, gender, years of experience, subspecialty and practice setting. Perception and readiness questions concerned viewpoints of diagnostic accuracy for AI, perceived benefits, apprehensions about job replacement and willingness to adopt AI in practice. Perception and readiness items were recorded on a 5-point Likert scale ranging from strongly disagrees to strongly agree. The survey targeted a sample of 100 practicing radiologists across different institutions in India, selected to ensure diversity in experience levels and subspecialties. The eligibility criteria of this study are limited to only active practice within diagnostic imaging and a minimum of one year of experience, thereby excluding radiologists who only exist in a research and education setting, allowing for more concentrated views on clinical insights. The questionnaire was administered through an electronic interface using a secure web portal with anonymous access for respondents. The response rate was optimized through periodic reminder emails biweekly within a data collection period of two months. Data analysis was with SPSS (Version 25.0).

## Descriptive statistics:

Mean and standard deviation and frequency distribution were generated for all demographic data and the responses of the survey.

## Inferential statistics:

T-tests and ANOVA to measure whether differences in perceptions and preparedness were significant for subgroups of demographic categories: Experience-level and subspecialty, at p < .05. Ethical approval was provided by the institutional review board for conducting the study and all the participants gave informed consent. The investigation maintained ethical standards by ensuring participant confidentiality, participation was at their will and the participants remained anonymous throughout the data gathering and analysis.

## Questionnaire:

The questionnaire has sections related to demographics such as age, years of experience and practice setting; familiarity with AI tools; perceived benefits of AI, like diagnostic accuracy and workflow improvement; concerns, such as job security and data privacy; ethical and regulatory viewpoints; and willingness to cooperate with AI developers. Moreover, the survey included open-ended questions to which participants could talk about particular concerns, expected challenges and suggestions for implementing AI in radiology. It offered some rich qualitative insights that were supported by quantitative data derived from the thematic analysis of open-ended questions. In this part of the survey, radiologists would voice unique perspectives or particular challenges that they anticipate about AI to provide depth to findings in the study. Table 1 below comprehensively summarizes the questionnaire, providing an overview of key focus areas and details included in each section.

## Results:

Table 2 outlines the levels of AI familiarity among respondents, reflecting the general AI literacy among radiologists. Table 3 shows radiologists' positive perceptions of AI's potential benefits, particularly in enhancing diagnostic accuracy and improving workflow efficiency. Table 4 highlights key concerns radiologists have about AI integration, such as job displacement, reliability and ethical implications. Table 5 presents radiologists' readiness for AI integration, showing that those with fewer years of experience report higher readiness. Table 6 indicates a strong need among radiologists for formal training, with a majority expressing support for AI-specific education. Table 7 demonstrates the impact of AI on job satisfaction among radiologists, revealing a positive perception for almost half of the participants, though some report concerns. Table 8 indicates significant ethical concerns among radiologists, particularly about accountability, transparency and data security. Table 9 reflects radiologists' familiarity with AI tools, showing that image analysis is well-known, while fewer are aware of broader AI applications. Table 10 highlights optimism among radiologists for AI's role in improving patient outcomes, with many anticipating improvements in diagnostic accuracy and patient care. Table 11 indicates strong interest among radiologists for structured training on AI's role in diagnostic imaging.

## Discussion:

According to the current study, there is an extraordinarily complex view of AI in diagnostic imaging among radiologists. The scientists demonstrate enthusiasm toward its potential, along with certain reservations that most possess. The majority of respondents have answered that AI promotes diagnostic accuracy and improves workflow efficiency. This conclusion is consistent with earlier studies, which indicate that AI can help radiologists streamline routine procedures and avoid diagnostic errors [8, 9]. Automation of some processes will leave AI free to speed and improve the accuracy of diagnostic results, which benefits patients. For the radiologists it means that they will face more complicated cases and deal less with routine image reviewing [10]. Despite AI's promises, there seem to be huge barriers with its implementation, as the physician's surveyed think. Job replacement was the most serious threat perceived since nearly half of those surveyed were concerned AI may eventually replace parts of the work of radiology. This may be exaggerated but it does reflect an understandable concern in the medical profession about how AI will affect job security. More experienced respondents reported they were not nearly as prepared to accept AI and this could suggest a familiarity with the more traditional methods of diagnosis contributes to resistance to technological change [11, 12]. Another issue would be the reliability whereby 42.9% of the respondents doubted the dependability of AI in a diagnostic situation. The algorithm was programmed to provide maximum precision, but it is not 100% and can deliver misdiagnosis sometimes. Misinterpretation or over dependency on the AI's output without enough human supervision might lead to potential catastrophic mistakes in diagnosis, thus negatively impacting the patient's care [13]. To minimize such risks, radiologists emphasized the fundamental necessity of human intervention in AI-based diagnostic tasks. Many radiologists have an opinion that AI is to be used as an augmentative tool and not as a replacement for diagnostics. Such an opinion reflects the "human-in-the-loop" model wherein AI can assist but the final decision will be taken by the radiologist alone. Another challenge highlighted by the respondents was the ethical issues. Radiologists questioned whether or not it was appropriate to assign an algorithm with the responsibility of making decisions in diagnostics and whether it is acceptable to leave sub-tell signs of complex cases unnoticed by AI [14- 15]. Then, the involved ethics are transparency concerning how AI makes the decisions and accountability if there is a diagnostic error. There is also a need for guidelines and standard regulation on AI utilization in clinics because without that, it means no clear definition of the layers of ethics concerned [16, 17-18]. Overall, respondents recognized AI's significant impact on the radiology profession, viewing it as an opportunity (61 %, 141 out of 232) rather than a threat (18 %, 42 out of 232), with a majority expressing belief in AI's relevance to future radiologists' career choices (60 %, 139 out of 232) [19]. Radiology residents generally hold a positive attitude toward AI, with 29.90% (1096/3666) agreeing that AI may reduce the demand for radiologists, 72.80% (2669/3666) believing AI improves disease diagnosis and 78.18% (2866/3666) feeling that radiologists should embrace AI [20]. A majority of respondents were positive/somewhat positive towards AI in radiography *e.g.*, 77.9 % (n = 342) thought that AI would have a positive effect on the profession and 26% thought that AI would reduce the administrative workload [21]. The results indicated that radiographers struggled to obtain AI-related education and training. This difficulty is exacerbated because the radiographers have noted a shortage of post-qualification education courses [22].

## Preparedness:

The younger and relatively less experienced radiologists are somewhat positive about the integration of AI and could be because they are closer to technology and adaptable to new technologies. These results indicate that early exposure to AI within the curriculum can likely lead to a more positive attitude toward the adoption of AI from future generations of radiologists. Such a pressing need is also underlined by the 54.8% who strongly agreed in the necessity for formal AI training programs to support safe and effective utilization of AI in radiology. Training programs that cover AI fundamentals, diagnostic applications and ethical considerations could prepare radiologists with knowledge and competence to work safely and responsibly alongside AI technology. To begin with, the communication that emphasizes Job-related issues might allay some fears such as the notion of the switch being a total replacement for radiologists by AI. More importantly, developing extensive training programs that are designed in light of radiologists' needs can help them understand and prepare themselves for applying AI technology. Thirdly, there is a need to define regulatory standards and ethical frameworks that define AI's role and accountability in practice in order to address ethical considerations related to the integration of AI into healthcare services.

## Conclusion:

While AI holds great promise for advancing diagnostic imaging, its successful integration into radiology requires more than technological progress. Addressing radiologists' perceptions, providing comprehensive training and establishing ethical guidelines are essential for fostering a human-AI partnership. As the field evolves, efforts must focus on preparing radiologists to collaborate with AI, leveraging its potential to enhance patient outcomes while preserving the unique value of human expertise.

## Figures and Tables

**Table 1:** Summary of the questionnaire

**Section**	**Focus Area**	**Details Included**
Demographics	Background information	Age, years in practice, practice setting
Familiarity with AI	Level of understanding of AI concepts	How familiar are you with machine learning and deep learning in radiology?
Perceived Benefits of AI	Views on AI's impact on diagnostic imaging	How beneficial do you believe AI is for improving diagnostic accuracy?
Concerns About AI	Potential challenges with AI integration	What concerns do you have regarding AI in radiology?
Ethical and Regulatory Perspectives	Attitudes towards data security and ethics	Do you feel there is a need for regulatory guidelines for AI use in radiology?
Readiness for AI Adoption	Openness to incorporating AI in practice	How willing are you to adopt AI tools in your daily practice?
Training and Education	Interest in additional AI training	Would you be interested in further training on AI applications in imaging?
Open-Ended Responses	Additional insights from radiologists	Please share any specific concerns or challenges you foresee with AI integration.

**Table 2:** Familiarity with AI

**Variable**	**Percentage (%)**
Age 25-35	34.5
Age 36-45	39
Age 46-55	20.5
Age 56+	6
< 5 Years' Experience	17
5-15 Years' Experience	46
> 15 Years' Experience	37
Familiar with AI	59
Unfamiliar with AI	41

**Table 3:** Perceived benefits of AI

**Perceived Benefit**	**Percentage (%)**
Enhanced Diagnostic Accuracy	65.5
Improved Workflow Efficiency	63
Reduction in Diagnostic Errors	59
Allows Focus on Complex Cases	54.5

**Table 4:** Barriers to AI integration

**Barrier**	**Percentage (%)**
Job Displacement	49
Reliability Concerns	43.5
Ethical Implications	40
Lack of AI Training	32.5

**Table 5:** Readiness for AI based on experience level

**Years of Experience**	**Ready for AI (%)**	**Not Ready for AI (%)**
< 5 Years	74	26
5-15 Years	59	41
> 15 Years	36.5	63.5

**Table 6:** Need for AI training programs

**Training Need**	**Percentage (%)**
Strongly Agree	55
Agree	27
Neutral	11.5
Disagree	6.5

**Table 7:** Perceived impact of AI on job satisfaction

**Perception of Job Impact**	**Percentage (%)**
Positive Impact on Job Satisfaction	49
Negative Impact	28.5
Neutral	22.5

**Table 8:** Ethical concerns regarding AI use in radiology

**Ethical Concern**	**Percentage (%)**
Accountability for Errors	63
Transparency in AI Algorithms	56
Data Privacy and Security	49
Bias in AI Systems	42

**Table 9:** Familiarity with AI tools among radiologists

**AI Application**	**Familiar (%)**
Image Analysis	63.5
Workflow Automation	42
Predictive Analytics	36.5
AI-Assisted Reporting	30

**Table 10:** AI's perceived impact on patient outcomes

**Perceived Patient Outcome Impact**	**Percentage (%)**
Improved Diagnostic Accuracy	65.5
Enhanced Patient Care	54
Reduction in Diagnostic Delays	48
Minimal or No Impact	13.5

**Table 11:** Interest in AI training programs

**Interest in AI Training**	**Percentage (%)**
Very Interested	54
Interested	30.5
Neutral	10
Not Interested	5.5
